# Editorial: Biomarkers for prognosis of neuroinflammation and neurodegeneration associated with acute and chronic viral diseases

**DOI:** 10.3389/fnins.2024.1354409

**Published:** 2024-01-16

**Authors:** Otávio de Melo Espíndola, Juliana Echevarria-Lima, Philippe V. Afonso

**Affiliations:** ^1^Instituto Nacional de Infectologia Evandro Chagas, Fundação Oswaldo Cruz, Rio de Janeiro, Brazil; ^2^Department of Immunology, Instituto de Microbiologia Paulo de Góes, Universidade Federal do Rio de Janeiro, Rio de Janeiro, Brazil; ^3^Unité Epidémiologie et Physiopathologie des Virus Oncogènes, Institut Pasteur, Université Paris-Cité, CNRS UMR 3569, Paris, France

**Keywords:** biomarkers, central nervous system, neuroinflammation, neurodegeneration, virus infection, prognostic markers, blood-brain-barrier

Many RNA and DNA viruses exhibit neuroinvasive properties and can be associated with acute or chronic neurological manifestations (Debiasi and Tyler, 2004). Therefore, rapid identification of pathogens causing central nervous system (CNS) disorders is crucial, and prognostic biomarkers are helpful for early disease management and follow-up of therapeutic interventions. However, studying biomarkers of neurodegenerative and neuroinflammatory processes related to viral infections is challenging due to the limited number of experimental models, difficulty in accessing the human CNS, and brain tissues being available generally postmortem (Rauf et al., 2022).

When studying neurodegenerative diseases triggered by viral infections, many factors should be considered. A factor that may potentialize CNS infection is the viral inoculum, which is too often disregarded. For instance, mouse models only develop neurological symptoms when infected with high doses of virus Yellow Fever virus (YFV), suggesting that a certain plasma YFV concentration is necessary for neuroinvasion (Douam et al., 2017). The route of infection on the one hand is also crucial. Neural cells may be directly exposed such as the olfactory cells, as described for human beta-coronaviruses (Desforges et al., 2014). Moreover, neurons may be infected through neuron-to-neuron transfer, as shown for herpes simplex and rabies viruses (Ugolini, 2011). Finally, neurons may be infected by blood-borne virus after bypassing the blood-brain-barrier (BBB), for example through infection of BBB endothelial cells, or transmigration of infected leukocytes in a “Trojan horse” strategy, as observed for Nipah virus (Mathieu et al., 2011), HTLV-1 (Afonso et al., 2008), and flaviviruses (Maximova and Pletnev, 2018). Some viruses can be highly neurotropic to immature CNS, such as the Zika virus (Garcez et al., 2016; Schuler-Faccini et al., 2016). Moreover, the site of infection may also be key in the development of symptoms. In this sense, Sha and Chen, at the Chongqing Three Gorges University, China, reviewed aspects of infection by all seven coronaviruses with the ability to infect humans, including routes of infection, neuroinvasion mechanisms, structural properties of viral proteins associated with virulence, and potential drug inhibition pathways.

Biomarkers for neurological involvement in viral infections often overlap with those of classical neurodegenerative diseases, such as Alzheimer's and Parkinson's diseases, multiple sclerosis, and amyotrophic lateral sclerosis. These include cerebrospinal fluid (CSF) and serum levels of Tau protein, neurofilament light and heavy chain proteins, intrathecal IgG production, astrocytic activation markers, and factors associated with microglial activation (Gomes et al., 2022; Gaur et al., 2023; O'Day, 2023). However, there are differences when considering acute infection and long-term progression, and the mechanisms may be distinct. While it seems easier to recapitulate acute diseases, one has to be more careful when considering long-progressing diseases. In particular, the many correlations between markers and diseases may not evidence a direct role of these elements. For instance, HTLV-1-associated myelopathy/tropical spastic paraparesis (HAM/TSP) richly illustrates a virus-induced long-term progressive neurodegenerative disease. HAM/TSP presents with motor disability and sensory, urinary, intestinal, and sexual disturbances. This clinical picture results from the continuous neuronal damage and gliosis of the thoracic and lumbar tracts of the spinal cord due to the response of cytotoxic CD8^+^ T-cells (CTLs) against infected CD4^+^ T-cells at sites of inflammation (Souza et al., 2021). Therefore, biomarkers for HAM/TSP development or progression rely mostly on inflammation markers (Tamaki et al., 2019; Souza et al., 2021; Freitas et al., 2022; Gomes et al., 2022). Silva De Castro et al. in a collaboration between the Federal University of Rio de Janeiro, Brazil, and the National Cancer Institute, Bethesda, MD, United States, investigated the effect of HTLV-1 infection on the cellular prion protein (PrP^C^) expression by CD4^+^ T-cells. It was observed that PrP^C^ levels were lower in HTLV-1-infected cells as well as the frequency of PrP^C+^ CD4^+^ T-cells, and it was induced in an IFN type II dependent manner by the HTLV-1 Orf I encoded p12 protein, which is associated with HTLV-1 persistence by favoring cell proliferation and the immune escape by promoting MHC class I degradation. Therefore, these results open a new direction in the investigation of the potential role of PrP^C^-associated pathways in the neuropathogenesis of HTLV-1 infection.

Biomarkers might also have distinct predictive values considering the site of sample collection. Proteomic profiles and biomarker concentrations differ between the ventricular and lumbar CSF compartments (Rostgaard et al., 2023). Therefore, one should pay attention to the CNS-affected area. Sensing or responding to immune signals such as interferons (IFN) may also be divergent when considering the cortex, the spinal cord, or other regions, posing the problem of viral replication and antiviral responses. Telikani et al. at La Trobe University and the University of Sydney, Australia, reviewed CNS antiviral mechanisms, discussing different immune signatures according to the responsiveness of cell types or subtypes, tissue location, and differentiation stage. This review presents the main response mechanisms of CNS resident cells against RNA and DNA viruses, highlighting the role of astrocytes as the main IFN-β producers in the brain, innate immune responses performed by microglial cells, the importance of autophagy by neuronal cell populations in the control of viral replication, and the role of glial cells in also promoting neuronal survival.

And finally, there is always a sweet spot between efficient intrathecal immune response and excessive inflammation. Despite the benefits of viral clearance, removal of cellular debris, and tissue repair, the consequences of neuroinflammation are primarily responsible for morbidity and mortality (Wyss-Coray and Mucke, 2002). Rocamonde et al. at the Université Lyon, France, reviewed the role of cytokine mediators in the development of inflammatory diseases in the CNS, the interactions between viruses and glial cells, and the mechanisms involved in exacerbated glial responses.

Overall, collaborative efforts of distinct research groups have helped to identify prognostic biomarkers and the mechanisms behind neurological involvement associated with distinct viral infections, yet many remain unclear. This Research Topic covers a range of original articles and reviews that discuss the current knowledge over the impact of viral infections on the CNS, highlighting the influence of routes of infection, target cells, mechanisms of immune response promoted by CNS resident cells, and the modulation of cellular functions. We believe that information organized in this Research Topic might contribute to support future investigations of biomarkers for CNS degeneration and inflammation associated with viral infections.

## Author contributions

OE: Writing—original draft. JE-L: Writing—review & editing. PA: Writing—original draft, Writing—review & editing.
